# The AhR/IL-22 axis in chronic gut inflammation: unraveling mechanisms and therapeutic prospects

**DOI:** 10.3389/fimmu.2025.1668173

**Published:** 2025-09-12

**Authors:** Huimin Kang, Zheng Chen, Baodong Wang, Zhiyun Chen

**Affiliations:** ^1^ The First Affiliated Hospital of Zhejiang Chinese Medical University (Zhejiang Provincial Hospital of Chinese Medicine), Hangzhou, China; ^2^ Zhou Hengde Provincial Famous Chinese Medicine Expert Studio, Hangzhou, China

**Keywords:** the AhR/IL-22 pathway, chronic intestinal inflammation, immune response, AhR, IL-22

## Abstract

Chronic inflammatory bowel diseases, including Crohn’s disease (CD), ulcerative colitis (UC), and post-infectious irritable bowel syndrome (PI-IBS), are characterized by immune-mediated intestinal inflammation and epithelial barrier dysfunction. Research indicates that the aryl hydrocarbon receptor (AhR)/interleukin-22 (IL-22) pathway is critical for intestinal homeostasis. This pathway can be activated by ligands from dietary and microbial sources (such as tryptophan metabolites), and AhR signaling in immune cells (particularly type 3 innate lymphoid cells (ILC3s) and T cells) is the primary driver of IL-22 production. IL-22 protects the intestinal barrier and regulates inflammatory responses by promoting epithelial repair, enhancing mucus and antimicrobial defenses, and strengthening tight junctions. Dysregulation of this pathway plays a key role in the pathogenesis of chronic intestinal inflammation, leading to exacerbated inflammatory processes and mucosal damage. Given its central role in barrier defense and repair, targeting the AhR/IL-22 pathway has emerged as a novel therapeutic direction for restoring intestinal homeostasis. This review summarizes the mechanisms of action of this pathway in chronic intestinal inflammation and explores its potential as a novel therapeutic target.

## Graphical Abstract

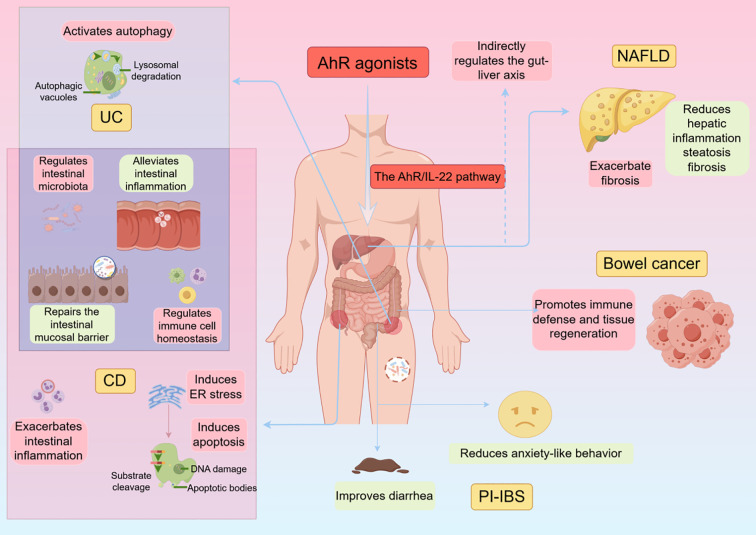

## 1 Introduction

Chronic intestinal inflammatory disorders, encompassing UC, CD and PI-IBS, represent a group of persistent and recurrent conditions that profoundly impact patients’ quality of life. These disorders are pathologically defined by sustained inflammatory responses within the intestinal mucosa, with primary clinical manifestations including the recurrent onset and remission of symptoms such as abdominal pain, diarrhea, weight loss, and fatigue (1–3). Inflammatory bowel disease (IBD) affects approximately 7 million people worldwide (4). Since the beginning of the 21st century, the morbidity of IBD has remained high in Western countries, while its incidence has surged in newly industrialized countries in Asia, Africa, and South America, becoming a major global public health challenge (5). High-income countries such as North America and Western Europe have the highest prevalence of IBD, while the incidence is rapidly increasing in low- and middle-income countries (6, 7). This indicates that the burden of IBD varies significantly across different regions and countries as socioeconomic development levels increase (7). Although the incidence of IBD in high-income countries tends to stabilize or decrease, its high prevalence and social burden remain a significant public health issue (8). Although current therapeutic approaches, including immunosuppressants, biologics, and agents targeting intestinal flora modulation, demonstrate efficacy in certain patients, a substantial proportion exhibit poor responsiveness to these treatments, and prolonged therapy may be associated with adverse effects (9, 10). Consequently, the identification of novel therapeutic targets and strategies is imperative to enhance the prognosis for individuals afflicted with chronic intestinal inflammation.

In recent years, significant attention has been directed towards elucidating the roles of tne AhR/IL-22 pathway in modulating intestinal immune responses and maintaining epithelial barrier integrity (11–13). The AhR functions as a ligand-activated transcription factor that is responsive to environmental cues, immune signaling, and cellular metabolic processes (14, 15). IL-22, a cytokine produced by specific immune cells, is instrumental in protecting the host from inflammatory damage in the gut by promoting the production of antimicrobial peptides and enhancing epithelial barrier function (16, 17). A growing body of evidence indicates that the AhR/IL-22 pathway is crucial for sustaining intestinal homeostasis and defense mechanisms. Upon activation, this pathway promotes the proliferation and differentiation of intestinal epithelial cells, enhances the integrity of the mucosal barrier, and regulates inflammatory responses, thereby offering new therapeutic opportunities for the management of chronic intestinal inflammation (14, 18, 19). Consequently, pharmacotherapeutic strategies targeting the AhR/IL-22 pathway have emerged as a central focus in the research of chronic intestinal inflammation, offering promising potential for therapeutic intervention.

## 2 Biological basis of the AhR/IL-22 pathway

AhR is an essential ligand-activated transcription factor that is part of the basic helix-loop-helix (bHLH)-Per-Arnt-Sim (PAS) family, and it is widely expressed in both immune and non-immune cells (20). The primary intracellular role of AhR is to detect small environmental molecules, facilitated by its ligand-binding domain (LBD), which possesses a unique conformation capable of binding a diverse range of ligands (21, 22). These ligands include both exogenous and endogenous substances, such as environmental pollutants, dietary compounds, and tryptophan metabolites (23). In the absence of ligands, AhR is typically localized in the cytoplasm, where it forms complexes with molecular chaperones like heat shock protein 90 (HSP90), X-associated protein 2 (XAP2), and p23 (24). Upon ligand binding, AhR translocates to the nucleus and forms a heterodimer with the Aryl Hydrocarbon Receptor Nuclear Translocator (ARNT), subsequently binding to specific DNA sequences (25, 26).

**Figure 1:**
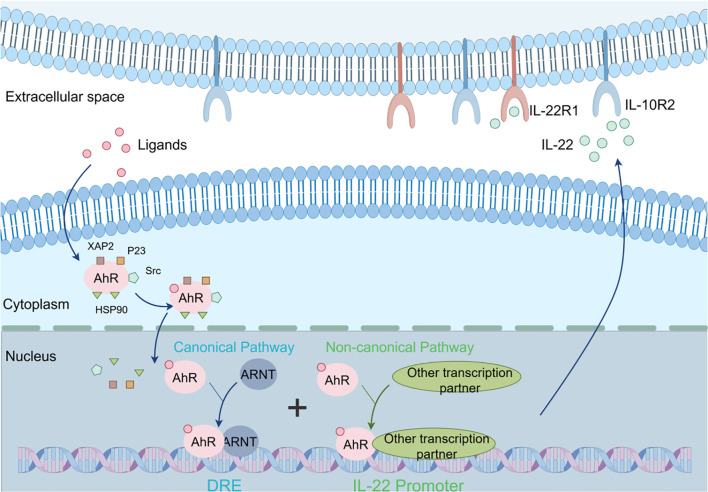
Cellular mechanisms by which AhR affects IL-22 production. In the absence of ligands, AhR is typically localized in the cytoplasm, where it forms complexes with molecular chaperones such as HSP90, XAP2, and p23. In the canonical pathway, after AhR ligands bind to AhR, AhR enters the nucleus and forms heterodimers with ARNT. These heterodimers bind to the DRE element in the promoter region of the IL-22 gene, initiating transcription of the IL-22 gene, ultimately producing IL-22 protein. In the non-canonical pathway, after AhR ligands bind to AhR, AhR can form complexes with transcription factors such as NF-κB and RORγt. These complexes collectively act on the promoter region of the IL-22 gene, promoting the production of IL-22. AhR, Aryl Hydrocarbon Receptor; DRE, Dioxin Response Element; IL-22R1, Interleukin-22 Receptor 1; IL-10R2, Interleukin-10 Receptor 2; ARNT, Aryl Hydrocarbon Receptor Nuclear Translocator; XAP2, Xenobiotic-Associated Protein 2; P23, Protein 23; HSP90, Heat Shock Protein 90.

Activation of AhR can affect IL-22 production (**Figure 1**). In the canonical pathway, AhR: ligand: ARNT trimer binds to the dioxin response element (DRE) upstream of the AhR target gene regulatory region in the cell nucleus, thereby regulating gene transcription, including IL-22 (27). ILC3 is the main IL-22-producing cell, and activation of the AhR in ILC3 promotes IL-22 secretion (28). The AhR can control ILC3s proliferation and turnover by up-regulating the Kit and Notch pathways to manage the proliferation, differentiation, and turnover of ILCs, thereby maintaining the stability of the ILC cell pool to regulate IL-22 production (29). The activation of AhR facilitates the production of IL-22 by influencing monocytes and naïve CD4+ T cells, which subsequently differentiate into Th17/22 cells (30–32). Furthermore, AhR modulates IL-22 production by regulating the function of various immune cells that indirectly impact T cell activity and IL-22 synthesis (33). In non-canonical pathways, after AhR ligands bind to AhR, AhR can form complexes with transcription factors such as nuclear factor kappa-light-chain-enhancer of activated B cells (NF-κB) and retinoic acid-related orphan receptor gamma t (RORγt). These complexes act together on the promoter region of the IL-22 gene to promote IL-22 production. Research also indicates that AhR not only independently regulates IL-22 production but also interacts with other signaling pathways. For instance, the Notch signaling pathway has been shown to augment IL-22 production in CD4+ T cells, contingent upon AhR activation (34). Additionally, interleukin-21 (IL-21) enhances IL-22 production by activating signal transducer and activator of transcription 3 (STAT3), which influences the epigenetic configuration of the IL-22 promoter and its interaction with AhR (35). Moreover, AhR activation elevates the expression of anti-inflammatory cytokines such as IL-22 and IL-10, while concurrently reducing the production of pro-inflammatory cytokines, including IFN-γ, IL-6, and TNF-α (20).

IL-22 acts by binding to its receptor, which is a heterodimeric receptor complex consisting of the subunits interleukin-22 receptor 1 (IL-22R1) and IL-10R2 (36). The expression of IL-22R1 is mainly localized to non-immune tissues like the skin, lungs, small intestine, liver, colon, kidneys, and pancreas, whereas IL-10R2 is widely found in immune cells (37). When IL-22 binds to its receptor, it activates STAT3 through the JAK (Janus Kinase)-STAT pathway pathway and also triggers STAT1 (38). Additionally, the MAPK (Mitogen-Activated Protein Kinase) pathway and the PI3K (Phosphatidylinositol 3-Kinase)-AKT (Protein Kinase B)-mTOR pathway are stimulated by IL-22 (39, 40). Through these signaling pathways, IL-22 plays a critical role in mucosal barrier immunity, tissue regeneration, and epithelial cell survival/proliferation. It further promotes the remodeling and repair of various tissues and organs, thereby sustaining the intrinsic host defense mechanisms that control pathogen invasion (40–42).

## 3 The role of the AhR/IL-22 pathway in intestinal homeostasis

### 3.1 The pathway of AhR/IL-22 signaling in the intestine

**Figure 2:**
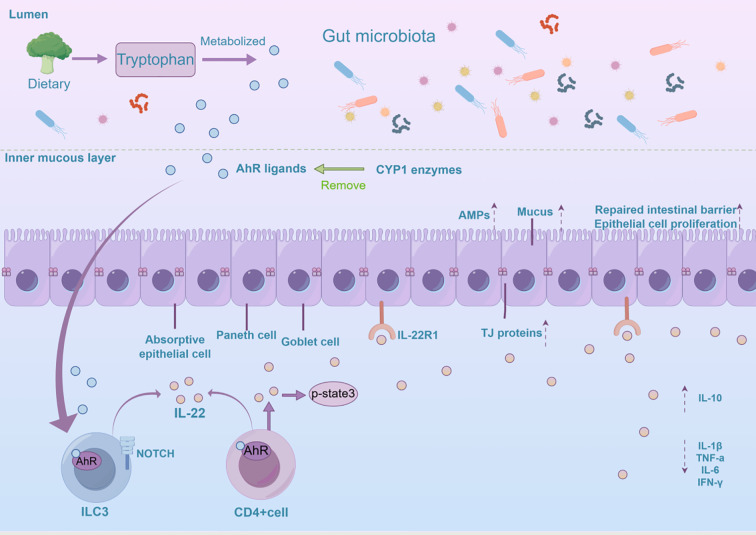
The mechanistic function of the AhR/IL-22 pathway in sustaining intestinal homeostasis is explored. AhR is activated in the intestine by various ligands, including tryptophan metabolites, microbiota-derived metabolites, and dietary compounds. This activation influences IL-22 expression in CD4+ T cells and ILC3s. IL-22 then promotes intestinal epithelial cell proliferation, tissue repair, and antimicrobial peptide secretion, maintaining the mucosal barrier, reducing inflammation, and supporting intestinal balance and homeostasis. ILC3, Group 3 Innate Lymphoid Cells; TJ protein, Tight Junction Protein; CYP1 enzymes, Cytochrome P450 enzymes; Amps, Antimicrobial Peptides.

Signaling through the AhR/IL-22 pathway in intestinal epithelial cells involves a very complex process (**Figure 2**). In the gut, AhR regulates IL-22 production through interactions with various signaling pathways. The classical AhR/IL-22 signaling pathway in the intestine mainly regulates the expression of target genes such as IL-22 by ligand activation of AhR, nuclear translocation, and heterodimerization with ARNT, thereby maintaining intestinal barrier function and immunization homeostasis (43, 44). Gut microbes synthesize tryptophan derivatives, which act as ligands for AhR and can activate the AhR signaling pathway, promoting IL-22 secretion (45). AhR plays an important role in regulating immune cell homeostasis in the intestinal tract, particularly in the maintenance of ILC3s and IL-22-producing ILC22 cells (46, 47). In addition, AhR aids in the production of IL-22 by CD4+ T cells and monocytes in the intestinal lamina propria (48). AhR signaling is also required for the maintenance of IL-22 production by intraepithelial lymphocytes (IELs) (49). Non-classical signaling pathways involve the synergistic interaction between the AhR and NF-κB signaling pathways, as well as interactions with other transcription factors, which play an important role in regulating intestinal immune response and inflammation.The AhR signaling pathway can synergize with the Toll-like receptor (TLR)/NF-κB signaling pathway to jointly promote IL-22 production (50). Short-chain fatty acids (SCFAs), such as butyric acid, also promote the expression of AhR and hypoxia-inducible factor 1 alpha (HIF1α) through the inhibition of inhibition of histone deacetylases (HDAC) enzymes and activation of G protein-coupled receptor 41 (GPR41), which in turn enhances IL-22 production (51, 52). The TGF-β signaling pathway is thought to play a role in regulating IL-22 production, especially in the context of intestinal tumorigenesis (53). Additionally, in the gut, AhR activity is regulated by cytochrome P450 enzymes (CYP1). CYP1 enzymes play an important role in regulating IL-22 production by metabolically removing AhR ligands, which, in turn, provides feedback regulation of the AhR signaling pathway (54). IL-22 shapes the composition and function of the intestinal microbiome, which can also lead to increased AhR signaling (55). These feedback mechanisms are crucial for preserving the balance of the intestinal immune system. IL-22, once produced, attaches to intestinal epithelial cells through its receptor, IL-22R1, and triggers downstream signaling pathways. For example, activation of the downstream STAT3 signaling pathway promotes epithelial cell proliferation, differentiation, and production of antimicrobial proteins (56). Thus, it plays a crucial role in maintaining intestinal barrier function and regulating intestinal immunity.

### 3.2 The function of the AhR/IL-22 pathway in the intestine

The intestinal epithelial barrier’s integrity is crucial for the gut’s physiological function. Any impairment of this barrier may result in increased intestinal permeability, which can contribute to a range of disorders, including inflammatory bowel disease, allergic reactions, and metabolic diseases (57, 58). The intestinal epithelial barrier serves a critical function in inhibiting the translocation of deleterious substances, including bacteria and toxins, across the intestinal mucosa into adjacent tissues, organs, and the systemic circulation (59). It also inhibits the growth of harmful microorganisms, recognizes and removes invading pathogens while ensuring intestinal immune tolerance (60, 61).

The AhR/IL-22 signaling pathway is crucial for maintaining intestinal homeostasis by preserving mechanistic barriers, modulating immune responses, and fostering microbial symbiosis within the gut (62). In the context of IBD, dysregulation of the AhR/IL-22 pathway can result in aberrant immune responses, thereby exacerbating disease progression (20). This pathway contributes to the amelioration of intestinal barrier dysfunction by enhancing tight junction integrity and reducing epithelial permeability. Activation of the AhR/IL-22 pathway regulates the expression of tight junction proteins, such as claudin and occludin, which are vital for maintaining the structural integrity and selective permeability of the intestinal epithelium (63). IL-22 plays a pivotal role in promoting the repair and maintenance of intestinal structure and function. During instances of intestinal injury or inflammation, IL-22 stimulates the activation, proliferation, and differentiation of intestinal stem cells, thereby expediting the restoration of the intestinal epithelium (64, 65). Moreover, IL-22 stimulates mucin synthesis and glycosylation in intestinal epithelial cells, thereby strengthening the mucosal barrier function (66).

The activation of AhR can modulate host immune responses by regulating gut microbial communities and promoting intestinal health (67, 68). Additionally, IL-22 has been shown to further impact the immune status of the gut by altering the composition of the gut microbiota (69). It influences the gut microbiota by affecting epithelial cell growth and differentiation, as well as facilitating mucus secretion and the production of antimicrobial proteins (55). Furthermore, microbial metabolites can enhance IL-22 production by activating AhR, establishing a positive feedback loop that bolsters the immune defense mechanisms of the gut (32). Research indicates that IL-22 expression is intricately linked to the diversity of intestinal microbiota, with IL-22 deficiency potentially resulting in dysregulation of the intestinal microbiota and contributing to conditions such as inflammatory bowel disease (20).

However, IL-22 does not invariably exert a protective effect. The role of IL-22 in IBD is complex. As mentioned earlier, under conditions such as impaired intestinal barrier function, infection, and inflammation, IL-22 can exert beneficial repair and defensive effects; however, in certain chronic inflammatory conditions and tumorigenesis, IL-22 may exacerbate the disease or promote tumor growth (66, 70, 71). In IBD, IL-22 promotes the chemotaxis of inflammatory cells and the expression of inflammatory factors, exacerbating the inflammatory reaction (66, 70). IL-22 can increase the expression of the tight junction protein Claudin-2, thereby increasing the permeability of the intestinal epithelium and reducing transepithelial electrical resistance (TEER), which may disrupt the integrity of the intestinal barrier and lead to excessive permeation of intestinal microorganisms (72, 73). Excessive IL-22 may also reduce the number of intestinal stem cells (ISCs), affecting the regenerative capacity of the intestinal epithelium, which may exacerbate intestinal mucosal injury in IBD (74, 75). IL-22 is involved in ER stress response (76, 77). IL-22 may enhance the growth and development of the intestine, as well as other tissues and neoplasms, by promoting angiogenesis (71, 78, 79). Consequently, the function of the AhR/IL-22 pathway in maintaining intestinal health and contributing to disease is multifaceted and complex.

## 4 The involvement of the AhR/IL-22 pathway in chronic inflammatory diseases of the intestinal tract

### 4.1 Ulcerative colitis

UC is a long-term inflammatory condition characterized by symptoms such as frequent diarrhea, mucus, abdominal pain, bloody stools, and pus in stools (80). The aetiology of UC is incompletely understood but has been linked to the interactions of genetic, environmental, and immune factors (80). Currently, clinically used medications for UC include 5-aminosalicylic acid analogs, corticosteroids, immunosuppressants, and biologics (81). In the intestinal tissues of UC patients, AhR expression is significantly reduced, which correlates with increased inflammation and impaired intestinal barrier function. AhR activation leads to an increase in IL-22 production, which inhibits inflammation and maintains intestinal homeostasis. And AhR agonists are able to attenuate symptoms of the dextran sulfate sodium (DSS)-induced colitis in model mice by upregulating the expression of IL-22, and this model is highly similar to human UC (82).

Increased intestinal permeability is one of the essential features of UC. Quercetin has been found to alleviate UC by activating AhR-mediated enhancement of tight junctions (TJs), thereby repairing intestinal barrier dysfunction (83). Indole-3-carbinol (I3C), a ligand of AhR derived from plants, exhibits promising therapeutic potential in the context of DSS-induced chronic colitis. I3C is posited to facilitate the restoration of epithelial integrity by enhancing the expression of tight junction proteins and reestablishing homeostasis within both the innate and adaptive components of the intestinal immune system. This effect is mediated through the downregulation of neutrophil and macrophage activity, alongside the modulation of Th17/Treg cell ratios (84). In a recent study using baicalein to treat UC in DSS-induced mice, it was found that baicalein improved symptoms and intestinal barrier function in mice with colitis by activating AhR, upregulating the expression of cytochrome P4501A1 (CYP1A1), promoting the production of IL-22 in ILC3,and enhancing the levels of tight junction proteins ZO-1 and occludin (85). Tryptophan supplementation-activated AhR-mediated induction of IL-22/Stat3 plays a crucial role in mucosal epithelial homeostasis and integrity (86).

Regulation of intestinal flora in UC involves the important AhR/IL-22 pathway. Amaranthus L.-derived exosome-like nanoparticle (PELN) treatment resulted in an increased abundance of Lactobacillus reuteri and indole derivatives, which activated AhR in conventional CD4+ T cells, leading to the down-regulation of Zbtb7b expression, differentiation of conventional CD4+ T cells into double-positive (DP) CD4+CD8+ T cells, and ultimately the attenuation of DSS-induced colitis in C57 mice (87). In DSS-induced UC in mice, atorvastatin can alleviate UC by regulating intestinal flora disorders, promoting microbial tryptophan metabolism, increasing the expression levels of AhR and IL-22, and further enhancing the expression levels of intestinal tight junction proteins such as ZO-1 and occludin (88). The combination of Rhizoma coptidis polysaccharides and berberine demonstrated more significant therapeutic effects by increasing the relative abundance of short-chain fatty acid (SCFA)-producing bacteria, which, in turn, elevated the level of SCFAs and activated the AhR/IL-22 pathway (89). A novel anti-colitis mechanism of oligofructose (FOS) promotes the production of indole-3-acetic acid (IAA) and indole propionic acid (IPA) to trigger AhR/IL-22 axis activation by alleviating intestinal dysbiosis and modulating microbial tryptophan metabolism (45). Fucoidan was able to ameliorate chronic colitis by promoting intestinal IL-22 expression, upregulating colonic IL-22 and fucosyltransferase 2 (FUT2) expression, and inducing IL-22 release from CD4+ T cells through the AhR pathway as well as IL-22 secretion by ILCs, thereby ameliorating luminal and mucosal flora disorders in the small intestine and colon (90). Akkermansia muciniphila (Akk) is a probiotic that reduces colonic inflammation by modulating tryptophan (Trp) metabolism to activate AhR signaling and up-regulate AhR target genes, including CYP1A1, IL-10, and IL-22 (91). Chen et al. used an *in vitro* lipopolysaccharide (LPS)-induced intestinal crypt epithelial cell (IEC-6) model and an *in vivo* DSS-induced UC mouse model to demonstrate that *Lactobacillus paracasei* L21 and its heat-inactivated postbiotic mitigated DSS colitis similarly attenuated DSS-colitis by modulating the NF-κB and HIF1α/AhR-IL-22-mucin 2 (MUC2) axes (92). High indole-3-lactic acid (ILA) production is a key tryptophan metabolic characteristic of L. plantarum which activated AHR downstream signaling (such as CYP1A1, IL-22, and STAT3) to alleviate colitis (93).

Furthermore, the AhR/IL-22 pathway is vital in regulating antimicrobial peptide secretion by intestinal epithelial cells. A specific degree of polymerization of chitosan (COS) can regulate tryptophan metabolism through the AhR/IL-22 pathway, attenuate colon injury and inflammation, down-regulate the levels of indoleamine 2,3-dioxygenase 1 (IDO1)—an enzyme that plays a key role in the tryptophan metabolism pathway—and restore the levels of tryptophan metabolites. Additionally, it promotes MUC2 expression and repairs the intestinal mucosal barrier (94). In the DSS-induced UC in mice, enhancing tryptophan metabolism associated with intestinal flora by reorganizing the structure of the intestinal flora can activate the AhR/IL-22 pathway, stimulate the phosphorylation of STAT3, increase the expression of antimicrobial peptides (AMPs) Reg3β and Reg3γ, limit bacterial colonization on mucosal surfaces, reduce bacterial translocation to protect the mucosa, and accelerate the proliferation of epithelial cells, thereby further restoring the structure and function of intestinal barriers (95). In addition, ISC (intestinal stem cell) regeneration was enhanced, and intestinal IL-22 secretion, along with its related transcription factor AHR, was increased in DSS-induced UC after L-fucose treatment. This treatment accelerated ISC proliferation and helped to heal the epithelial barrier through the activation of nuclear AHR, stimulating the secretion of IL-22 from CD4+ T cells in the splenocytes of mice and from ILC3 cells in the LPMCs (48). In a DSS-induced mouse organoid inflammation model, Hymenolepis nana antigens promote intestinal stem cell proliferation and differentiation through the AhR/IL-22 signaling pathway, thereby alleviating ulcerative colitis (96). In UC, the AhR/IL-22 pathway may also function through the activation of autophagy. The activation of autophagy not only helps to maintain the homeostasis of the intestinal epithelium but also attenuates the inflammatory response and reduces intestinal damage (97, 98). Therapeutic interventions targeting the AhR/IL-22 axis may offer novel avenues for the management of ulcerative colitis, potentially enhancing autophagic processes and improving intestinal health outcomes (99, 100).

The complexity of UC has historically hindered a comprehensive understanding of its etiology and the identification of drug targets. In the field of UC treatment, the AhR/IL-22 signaling pathway plays a multifaceted role: it promotes intestinal barrier repair, regulates immune responses, activates autophagy processes, modulates intestinal microbiota balance, and accelerates intestinal epithelial cell regeneration. These findings highlight the potential value of this pathway in UC treatment and provide important clues for developing novel therapeutic strategies and targets.

However, given the current research progress and limitations, existing studies primarily rely on cell experiments and animal models, lacking validation in clinical patients. It is recommended to conduct multicenter clinical trials to assess the safety and efficacy of AhR agonists in UC patients; or encourage researchers to explore the interactions between the AhR/IL-22 pathway and other signaling pathways to gain a more comprehensive understanding of the role of the AhR/IL-22 pathway in the pathogenesis of UC.

### 4.2 Crohn’s disease

CD is a multifaceted chronic inflammatory bowel disorder characterized by significant clinical symptoms such as abdominal pain, diarrhea, abdominal masses, and hematochezia (101). The precise etiology of Crohn’s disease remains incompletely elucidated; however, an increasing body of research indicates that interactions among genetic predispositions, dysbiosis of the gut microbiota, immune system dysregulation, and environmental influences are pivotal in the pathogenesis of the disease (102–106). Current therapeutic strategies for CD encompass aminosalicylic acid compounds (commonly known as 5-ASA agents), glucocorticoids, immunosuppressants, and biologic therapies (107). Although these treatments have been effective in enhancing patients’ quality of life and managing disease activity, concerns persist regarding their potential adverse effects and the safety associated with prolonged use (108, 109).

In CD, changes in the AhR/IL-22 pathway have attracted widespread attention. Studies have shown that AhR activation is closely associated with the pathological processes of CD (20, 110). The 2,4,6-Trinitrobenzolsulfonic acid (TNBS)-induced mouse model can mimic multiple pathological traits of Crohn disease, and 2,3,7,8-tetrachlorodibenzo-p-dioxin (TCDD), an environmental pollutant that mainly affects the human body through diet, can alleviate colon inflammation in the TNBS colitis mouse model by partially producing regulatory immune cells after activating AhR (111). In TNBS-induced colitis in humanized mice, the nontoxic AHR agonist methyl 2-(1’H-indole-3’-carbonyl)-thiazole-4-carboxylate (ITE) induced functional human Tregs, which inhibited effector T-cell proliferation *in vitro* in a CD39- and granzyme B-dependent manner, leading to the up-regulation of IL-22. This demonstrated that ITE promotes mucosal immune homeostasis and protects against the development of colitis (112). In patients with CD, Th17 cell activity is usually increased, leading to the overproduction of IL-22 (32). By activating AhR, certain endogenous ligands can promote IL-22 production and enhance Th17 cell activity (33). Infliximab (IFX) therapy markedly increased IL-22 mRNA expression in the intestinal mucosa of patients with CD. Furthermore, the inhibition of AhR significantly suppressed the differentiation of IL-22+CD4+T (Th22) cells induced by anti-tumor necrosis factor (TNF) treatment in these patients (113). In patients with CD, the expression levels of IL-22 are frequently modified, potentially influencing the integrity of the intestinal barrier and the modulation of the immune response (77). In a mouse model of TNBS-induced colitis, Higher levels of IL-22 were observed in dendritic cells (DCs) with AhR activation mediated by 6-formylindole [3,2-b] carbazole (FICZ) (114).

A protective function is played by IL-22 in CD to some extent by promoting the production of antimicrobial peptides and mucus that enhance intestinal barrier defense (66). IL-22 administration stimulates intestinal epithelial cells to upregulate the expression of tight junction proteins, such as claudin-1 and ZO-1, and enhances trans-epithelial resistance. This indicates that IL-22 plays a protective role in the intestinal mucosa of patients with CD by preserving the integrity of the epithelial barrier and mitigating inflammation (113). The activation of AhR enhances IL-22 production and affects the composition and function of the intestinal microbiota, which in turn influences the progression of CD (55). Ganoderic Acid A (GAA) can potentially ameliorate inflammatory bowel disease by modulating the intestinal flora and enhancing AhR activity, which in turn promotes IL-22 production and improves intestinal barrier function (115).

Despite the protective effects of IL-22, in the pathological state of CD, excess IL-22 may induce hyperproliferation of epithelial cells and the production of pro-inflammatory cytokines, further aggravating inflammation. Research has indicated that IL-22 might have a dual function in both acute and chronic inflammation in CD, aiding in the repair of epithelial cells while potentially worsening inflammation in certain situations (116). In patients with active Crohn’s disease CD, IL-22 induces an endoplasmic reticulum (ER) stress-responsive transcriptional program in colonic epithelial cells, leading to a disease response characterized by the induction of apoptosis. In contrast, the genetic ablation or antibody blockade of IL-22 mitigates the ER stress response and alleviates the disease, indicating a pro-inflammatory role for IL-22 in CD (77). Furthermore, excessive activation of the IL-22 pathway may exacerbate intestinal inflammation and potentially increase the risk of colon cancer (117).

In summary, the AhR/IL-22 pathway plays a multifaceted role in CD, encompassing various mechanisms such as modulation of the immune response, enhancement of intestinal barrier function, and influence on the microbiota balance. Although IL-22 may have a protective role, its overexpression can intensify inflammation in the pathological context of CD. Consequently, a comprehensive investigation into the roles of AhR and IL-22 in CD not only enhances the understanding of its pathomechanisms but also identifies potential targets for the development of novel therapeutic strategies.

### 4.3 Post-infection irritable bowel syndrome

Irritable Bowel Syndrome (IBS) is a prevalent functional gastrointestinal disorder, the pathogenesis of which may be associated with a range of factors, including visceral hypersensitivity, dysbiosis of the intestinal microbiota, dysfunction of the intestinal barrier, and low-grade inflammation. Its main symptoms include recurrent abdominal pain and changes in bowel habits, often accompanied by non-painful abdominal discomfort, anxiety, depression, and other psychiatric symptoms (118–120). After acute infectious diarrhea caused by bacteria, viruses, or parasites, 10%-30% of patients develop symptoms of IBS with a predominantly diarrheal condition known as PI-IBS (121). Low-grade inflammation in the gut and increased intestinal permeability have been associated with dysfunction of the AhR/IL-22 pathway, which may lead to increased intestinal sensitization and worsening of symptoms in patients with IBS. For example, mice with AhR-specific deficiency in macrophages are more susceptible to TNBS-induced IBS (122, 123). *Lactobacillus plantarum* D266 (Lp D266) can shape the gut microbiota and enhance tryptophan (Trp) metabolism, thereby activating AhR and subsequently enhancing IL-22 production to maintain gut homeostasis. In addition, the combined use of Lp D266 and Trp can synergistically improve IBS symptoms (124). Although the detailed pathological mechanisms of PI-IBS are unknown, recent studies have shown changes in the AhR/IL-22 pathway within preclinical models of PI-IBS.Research has found that *Lactobacillus rhamnosus* GG (LGG) can effectively prevent porcine epidemic diarrhea virus (PEDV) infection in piglets. The metabolites of Lactobacillus rhamnosus GG (LGG) interact with ILC3s in the jejunum of piglets through AhR. This interaction promotes the activation of ILC3s and the production of IL-22. Subsequently, IL-22 promotes the proliferation of porcine intestinal epithelial cell line J2 (IPEC-J2) cells and activates the STAT3 signaling pathway, thereby preventing PEDV infection (13). In a mouse model of PI-IBS, the AhR/IL-22 signaling pathway shows reduced expression, but administering IL-22 helps restore intestinal permeability and colonic sensitivity, enhances cognitive function, and lessens anxiety-like behavior (125). In PI-IBS, this pathway is closely related to the regulation of gut microbiota, immune response, and intestinal barrier function. The AhR/IL-22 pathway is anticipated to be a new target for addressing symptoms linked to post-infectious irritable bowel syndrome.

### 4.4 Other related diseases

#### 4.4.1 Non-alcoholic fatty liver disease

The progression of NAFLD is a result of a multifactorial interaction in which chronic inflammation, abnormal lipid metabolism, and dysregulation of the intestinal-hepatic axis combine to assist in the commencement and evolution of the disease (126–128). AhR is expressed at high levels in resting hepatic stellate cells (HSC) but decreases with HSC activation. In studies of human and mouse hematopoietic stem cells, we found that AhR can block the activation of hematopoietic stem cells and the expression of genes required for liver fibrosis. Developing non-toxic AhR agonists or strategies to activate AhR signaling in HSCs could be used to prevent or treat liver fibrosis (129). Short-chain fatty acids and other metabolites produced by gut microbes can regulate hepatic metabolic and inflammatory pathways through interactions with host cell receptors (130). AhR activation promotes β-oxidation of hepatic fatty acids and reduces hepatic fat accumulation, thereby counteracting the development of NAFLD. Activation of AhR promotes the production of short-chain fatty acids, which, in turn, enhances gut barrier function and reduces hepatic fat deposition and inflammatory response (82, 131). Supplementation with quercetin alleviates obesity by restoring the gut microbiota dysbiosis induced by HFD in obese mice, thereby increasing IPA levels to activate the AhR/IL-22 pathway, which enhances intestinal barrier integrity and suppresses chronic inflammation (132). Additionally, activation of AhR is capable of influencing the activity of macrophages and other immune cells, thereby modulating the inflammatory environment of the liver (133). The “gut-liver axis” is one of the key factors in the pathogenesis of NAFLD, transporting intestinal microbial metabolites and inflammatory mediators to the liver via the portal vein, and the AhR may be indirectly involved in the regulation of NAFLD by influencing the composition of the gut microbiota (134). By regulating the metabolism of the gut microbiota, AhR may also influence the immune environment of the gut, which, in turn, improves liver health (135).

IL-22 promotes intestinal health by regulating the function of intestinal epithelial cells and inhibiting lipid absorption, thus alleviating metabolic disorders associated with obesity to a certain extent (136). IL-22 signaling is inhibited by a high-fructose and high-fat diet, which may affect the health status of the liver endogenously. High-fat diets not only lead to low-grade chronic inflammation but also alter the gut microbiota, which in turn affects IL-22 production (137). Obesity and high-calorie diets rapidly inhibit IL-22 production, leading to impaired gut barrier function, which exacerbates the risk of metabolic diseases (138). IL-22 plays a protective role in the liver, promoting hepatocyte survival and proliferation through the activation of signaling pathways (e.g., the STAT3 pathway) in hepatocytes, thereby reducing hepatocyte injury caused by NAFLD. Studies have shown that IL-22 is significantly upregulated in patients with chronic liver disease and correlates with hepatocyte proliferation and the degree of inflammation (139, 140). IL-22 inhibits the production of pro-inflammatory cytokines, a role that is critical for reducing hepatic inflammation and promoting liver repair (141–143). By regulating metabolism-related signaling pathways such as AMPK, AKT, and mTOR, IL-22 not only improves the metabolic status of hepatocytes but also enhances the anti-apoptotic capacity of hepatocytes (143, 144). However, the role of IL-22 in liver fibrosis is dual: it promotes hepatocyte repair while potentially exacerbating fibrosis when overexpressed. This phenomenon may be related to the pro-inflammatory effects of IL-22, which can stimulate inflammatory responses and abnormal cell proliferation (145).

Nonetheless, there are fewer studies investigating the direct role of the AhR/IL-22 pathway in NAFLD, and its exact mechanism still needs further exploration. An in-depth study of this pathway’s mechanism may provide new insights for the treatment of NAFLD.

#### 4.4.2 Bowel cancer

The AhR/IL-22 pathway is also important in bowel cancer. This pathway plays a crucial role in immune defense and tissue regeneration in the intestine, with multiple effects such as pro-survival signaling, cell migration, developmental abnormalities, and angiogenesis (146). AhR can influence the makeup of the gut microbiota by regulating other cytokines and signaling pathways, which can further affect the immune status of the intestinal tract and tumor progression (147, 148). IL-22 enhances intestinal barrier function and promotes the production of antimicrobial proteins, which may be protective against the development of intestinal cancers in some cases (66). In cases of chronic inflammation, the activation of the AhR/IL-22 pathway might result in the unusual growth of intestinal epithelial cells and the development of tumor (149). Even though IL-22 is known for its protective effects on intestinal health, its contribution to the progression of intestinal tumors may be exploited by tumor cells, thereby promoting tumor growth and metastasis (146). In some cases, overexpression of IL-22 is associated with the progression of intestinal tumors, while in other cases, it may inhibit tumorigenesis by promoting epithelial cell repair and regeneration (116, 146). IL-22 expression is significantly higher in human colon cancer tissue than in healthy tissue and promotes tumor cell proliferation. In addition, IL-22 may also support tumor growth by promoting angiogenesis (71, 78).

## 5 Conclusions and perspectives

This review consolidates current knowledge on the mechanistic role and therapeutic applications of the AhR/IL-22 pathway in chronic intestinal inflammatory diseases. (**Table 1**, **Table 2**). The AhR/IL-22 signaling pathway plays a crucial role in the regulation of intestinal immunity, the maintenance of the mucosal barrier, and the modulation of inflammatory responses. Within the context of chronic intestinal inflammatory disorders, such as CD and UC, the activation of this pathway is intricately linked to the mitigation of inflammation and the stabilization of intestinal barrier function. Extensive research has demonstrated that this pathway’s activation can effectively promote the proliferation and differentiation of intestinal epithelial cells, enhance mucosal barrier integrity, and modulate the inflammatory response appropriately. Additionally, its active involvement in maintaining microbial balance within the intestines and influencing the host immune response underscores its potential as a foundation for developing innovative therapeutic interventions.

**Table 1:** The role of the AhR/IL-22 pathway in chronic intestinal inflammatory diseases and other related conditions.

Disease Mechanism	UC	CD	IBS	NAFLD	Bowel cancer
Maintaining intestinal epithelial cell integrity and function	+ (85, 88, 94)	+ (113)	+ (123, 125)	–	+ (66)
Enhances intestinal cell repair and regeneration	+ (48, 84, 99)	+ (66, 116)	–	–	+ (116, 146)
Promotes hepatocyte proliferation	–	–	–	+ (139, 140)	–
Secretion of antimicrobial peptides	+ (91, 94, 95)	+ (66)	–	–	+ (66)
Regulation of immune cell function	+ (85, 88, 91)	+ (32, 33, 112)	+ (122, 123)	–	–
Suppression of the inflammatory response	+ (85, 88, 94, 95)	+ (111, 114)	+ (122, 123)	+ (141–143)	–
Promotes inflammatory response	–	+ (77, 116, 117)	–	+ (145)	–
Modulation of the intestinal microbiota	+ (45, 87, 88, 90, 95)	+ (55, 115)	+ (125)	+ (134, 135)	–
Regulation of lipid metabolism	–	–	–	+ (129, 136)	–
Activation of cell death	–	+ (77)	–	–	–
Inhibition of cell death	–	–	–	+ (143, 144)	–
Modulation of central nervous system sensitivity	–	–	+ (125)	–	–
Abnormal proliferation of intestinal epithelial cells	–	–	–	–	+ (149)
Promotion of tumor invasion and metastasis	–	–	–	–	+ (146)

In **Table 1**, “+”indicates that published literature supports the effect; “-” indicates a lack of published literature to support the effect.

**Table 2:** Outlines the mechanisms of action and effects of various compounds and pharmaceuticals on the AhR/IL-22 pathway.

Name	Mechanism	Result	Reference
Chitooligosaccharides	Restores the AHR-IL-22 pathway to normal, and promotes MUC2 expression	Alleviates DSS-induced colitis in mice	(94)
Akkermansia muciniphila	Regulates Trp metabolism, activates AhR signaling and upregulates AhR target genes, such as IL-22	Reduces colon inflammation	(91)
Atorvastatin	Regulates intestinal flora imbalance, enhances microbial tryptophan metabolism, and increases AhR and IL-22 expression.	Alleviates UC	(88)
Dietary tryptophan supplementation	Activates the AhR-mediated IL-22/Stat3 pathway	Amelioration of DSS-induced colitis	(86)
Fructo-oligosaccharides	Regulates microbial tryptophan metabolism promotes the production of IAA and IPA, thereby triggering AhR/IL-22 axis activation	Reduces symptoms of DSS-induced colitis	(45)
Portulaca oleracea L-derived exosome-like nanoparticles	Increasing the abundance of Lactobacillus reuteri and raising the levels of indole derivatives leads to the activation of AhR in conventional CD4+ T cells and increases IL-22 levels.	Reduces UC in mice	(87)
*Lactiplantibacillus plantarum* D266	Shaping the gut microbiota and Trp metabolism leads to activation of AhR and subsequent enhancement of IL-22 production	Improvement of IBS symptoms	(124)
Baicalein	Upregulates CYP1A1 expression and promotes IL-22 production in ILC3 through activation of AhR	Ameliorates symptoms and intestinal barrier function in UC mice	(85)
Coptis chinensis polysaccharides and Berberine	Boosts SCFA-producing bacteria, raising SCFA levels and activating the AhR/IL-22 pathway	Improvement of symptoms in UC mice	(89)
L-Fucose	Stimulation of IL-22 secretion by CD4+ T cells in mouse splenocytes and ILC3 cells in LPMCs through activation of the nuclear AHR	Improvement of symptoms in UC mice	(48)
Fucoidan	Decreases UC-induced AhR and IL-22 expression	Treats with UC induced in rats	(90)
6-formylindolo [3,2-b]carbazole	AHR physiological activator, FICZ/AHR/CYP1A1 feedback regulation, stimulates IL-22 expression by a variety of different immune cells (including ILC3)	Enhances the reinforcement of the intestinal epithelial barrier and aids in tissue repair, supporting intestinal balance.	(62)
Lactobacillus rhamnosus GG	Promotes ILC3 activation and IL-22 production through AhR interaction with ILC3s	Prevention of virus enteric infections	(13)
Hymenolepis nana antigens	Enhances ISCs growth and development via the AhR/IL-22 pathway.	Alleviating symptoms in UC mice	(96)
Ganoderic acid A	Regulation of intestinal flora and enhancement of AhR activity to promote IL-22 production	Improvement of inflammatory bowel disease	(115)
Infliximab	Promotes IL-22+CD4+ T (Th22) cell differentiation in CD patients through AhR	improves CD symptoms	(113)
Dried ginger essential oil	Regulation of intestinal microbiota and tryptophan metabolite IAA-AHR/IL-22/STAT3 signaling axis	Alleviating 5-Fluorouracil-induced damage to the intestinal epithelial barrier in mice with mucositis	(12)
Lactobacillus paracaseiL21	Activates the HIF1α/AhR pathway, increases IL-22 and mucins MUC2 to restore the goblet cell population	Alleviates DSS-induced colitis	(92)
Quercetin	Increases IPA levels to activate the AhR/IL-22 pathway	Reduces obesity and chronic intestinal inflammation	(132)
Lactiplantibacillus plantarum producing high levels of indole-3-lactic acid	Activates AHR signaling in the intestine by metabolizing tryptophan, activating downstream AHR signaling (such as CYP1A1, IL-22, and STAT3) to alleviate colitis	Alleviates DSS-induced colitis in mouse models	(93)

Although the central role of the AhR-IL-22 axis in intestinal diseases is undeniable, further research is still needed to gain a deeper understanding of the pathological mechanisms underlying these diseases. While the role of AhR in immune regulation and maintaining intestinal homeostasis has become increasingly clear, the activation mechanisms of AhR in specific disease stages remain unclear. AhR has multiple ligands, whose sources and mechanisms of action are complex and diverse, making it extremely challenging to precisely elucidate the activation process of AhR (14, 150, 151). Additionally, the complex conversion mechanisms of IL-22 under pathological conditions require further exploration, as the molecular mechanisms and key influencing factors of its functional conversions remain poorly understood. This may be attributed to the high complexity of the intestinal microenvironment, where factors such as cell types, cytokine networks, and the intestinal microbiome may all influence IL-22’s function (70, 152, 153). Future research should investigate the precise activation mechanisms of AhR in the context of specific intestinal diseases, elucidating the key nodes and microenvironmental factors involved in IL-22 functional conversion.

Furthermore, although the AhR/IL-22 pathway has demonstrated significant therapeutic potential in basic research on chronic intestinal inflammatory diseases, its clinical application still faces numerous challenges. Current clinical trials are mostly in the preliminary exploratory phase, lacking clear definitions and strict application of treatment parameters, which limits the full realization of their clinical efficacy and may pose potential safety issues (14, 154, 155). To advance the clinical application of the AhR/IL-22 pathway, future research should prioritize high-quality clinical trials to accurately define treatment parameters, including optimal dosage, administration frequency, treatment duration, and patient selection criteria. Although the clinical application of the AhR/IL-22 pathway holds great promise, its advancement must be conducted with scientific rigor. Further research is necessary to develop more precise targeted interventions to drive progress in the treatment of intestinal diseases. In fact, the AhR/IL-22 pathway has emerged as a key regulatory pathway in intestinal diseases and represents a promising target for novel targeted therapeutic interventions in chronic inflammatory bowel diseases.
